# Diagnostic criteria and proposed management of immune-related endocrinopathies following immune checkpoint inhibitor therapy for cancer

**DOI:** 10.1530/EC-22-0513

**Published:** 2023-04-17

**Authors:** Ruth Percik, Sherwin Criseno, Safwaan Adam, Kate Young, Daniel L Morganstein

**Affiliations:** 1 Institute of Endocrinology, Diabetes and Metabolism, Sheba Medical Centre, Ramat Gan, Israel; 2 Department of Endocrinology, University Hospital Birmingham, Birmingham, UK; 3 Department of Endocrinology, The Christie NHS Foundation Trust, Manchester, UK; 4 Department of Endocrinology, Chelsea and Westminster Hospital, London, UK; 5 Royal Marsden Hospital, London, UK

**Keywords:** cancer, checkpoint inhibitor, immunotherapy, endocrinopathy, hypopituitarism, thyroid

## Abstract

Checkpoint inhibitors are now widely used in the management of many cancers. Endocrine toxicity is amongst the most common side effects. These endocrinopathies differ from most other immune-related toxicities in frequently being irreversible and rarely requiring cessation of checkpoint inhibitor therapy. This review considers an approach to the presentation and diagnosis of endocrinopathies, compared to classical endocrine diagnosis, suggesting improvements to classification and treatment based on fundamental endocrine principles. These will help to align management with other similar endocrine conditions and standardise the diagnosis and reporting of endocrine toxicity of checkpoint inhibitors to improve both endocrine and oncological care. In particular, the importance of considering any inflammatory phase (such as painful thyroiditis or hypophysitis resulting in the pituitary enlargement), from the endocrine consequences (transient hyperthyroidism followed by hypothyroidism, pan-hypopituitarism or isolated adrenocorticotrophic hormone deficiency), is highlighted. It is also important to consider the potential confounder of exogenous corticosteroids in adrenal suppression.

## Introduction

Endocrinopathies are amongst the most frequent adverse events of immune checkpoint inhibitors (ICPIs). In common with all other side effects of systemic anticancer treatment, they are currently reported according to the Common Terminology Criteria for Adverse Events (CTCAE, currently updated to version V1 for reporting and case management) (1). In this standardized grading system, symptoms’ severity and need for intervention determine the grading stratification (1 – mild, 5 – death) and guide decisions regarding continuation of immunotherapy and need for an immunomodulatory intervention (Table 1).

**Table 1:** CTCAEv5 classification of endocrine dysfunction.

	CTCAE					
	Toxicity	Grade				
		1	2	3	4	5
Thyroid	Hyperthyroidism	Asymptomatic; clinical or diagnostic observations only; intervention not indicated	Symptomatic; thyroid suppression therapy indicated; limiting instrumental ADL	Severe symptoms; limiting self-care ADL; hospitalization indicated	Life-threatening consequences; urgent intervention indicated	Death
	Hypothyroidism	Asymptomatic; clinical or diagnostic observations only; intervention not indicated	Symptomatic; thyroid replacement indicated; limiting instrumental ADL	Severe symptoms; limiting self-care ADL; hospitalization indicated	Life-threatening consequences; urgent intervention indicated	Death
Pituitary	Hypophysitis	Asymptomatic or mild symptoms; clinical or diagnostic observations only; intervention not indicated	Moderate; minimal, local or noninvasive intervention indicated; limiting age-appropriate instrumental ADL	Severe or medically significant but not immediately life-threatening; hospitalization or prolongation of existing hospitalization indicated; limiting self-care ADL	Life-threatening consequences; urgent intervention indicated	Death
	Hypopituitarism	Asymptomatic or mild symptoms; clinical or diagnostic observations only; intervention not indicated	Moderate; minimal, local or noninvasive intervention indicated; limiting age-appropriate instrumental ADL	Severe or medically significant but not immediately life-threatening; hospitalization or prolongation of existing hospitalization indicated; limiting self-care ADL	Life-threatening consequences; urgent intervention indicated	Death

However, endocrinopathies differ from other immune-related adverse events (irAEs) in several aspects. The long-term clinical manifestations stem from the hormone deficiency, whilst the inflammatory process itself may yield only minimal manifestations. As the hormone production of an involved gland ceases completely and irreversibly in most cases, lifelong hormone replacement should be initiated, whilst discontinuation of immunotherapy and/or immunomodulation with glucocorticoid therapy is usually unnecessary, with immunosuppressive doses of glucocorticoids only rarely required where the initial inflammation causes severe symptoms. Lastly, optimal exogenous correction of hormone deficiencies is readily available, based on an early and accurate diagnosis. Thus, even severe symptoms usually resolve rapidly after the initiation of hormone replacement.

These unique characteristics mean a classification based predominately on symptom severity at presentation is not helpful in guiding decisions about hormone replacement and continuation or discontinuation of ICPI therapy for endocrinopathies (2). Immune-related endocrinopathies do not fit into a five-tier symptoms severity stratification either diagnostically or therapeutically as they require binary diagnostic criteria based on the demonstration of absolute or relative hormone deficiency. Consequently, documentation and reporting of the incidence, severity and nomenclature of endocrinopathies (e.g. hypophysitis vs isolated adrenocorticotrophic hormone (ACTH) deficiency) are currently challenging. There might be an under-recognition of asymptomatic hormonal abnormalities that may still benefit from long-term treatment such as sub-clinical hypothyroidism. There are also implications for trial protocols, as endocrinopathies already present an exception, where current guidelines support the continuation of ICPIs, having initiated hormone replacement therapy, regardless of the grade. Furthermore, some endocrine irAEs require adjustment and correlation with standard endocrine diagnostic criteria (e.g. hyperglycaemia).

These recommendations complement published guidance, including emergency management guidelines endorsed by the Society for Endocrinology (3), and recent European guidelines (4). They extend these by addressing the requirement for a reliable classification, combined with an approach to the management of endocrine irAEs which will accurately reflect the clinical status, need for hormone replacement and ensure consistency with wider endocrine practice.

## General considerations for endocrine irAEs

The potential mechanisms underlying ICPI toxicity have been reviewed elsewhere (5). When compared to the classic, non-iatrogenic autoimmune endocrinopathies, ICPI-induced endocrinopathies (ICPI-IEs) differ both in their presentation and immune features. Although classic autoantibodies such as GAD-65 (6) and anti-TPO antibodies (7, 8) can be present, the literature to date suggests that they are less frequently detected than in classic autoimmune disease, suggesting a different mechanism of gland dysfunction. The timescale often differs as well, with rapid evolution into complete gland destruction frequently described with ICPI-IE (9, 10). In contrast to non-endocrine irAEs, the active autoimmune phase of endocrine irAEs is usually clinically silent excluding two exceptions, both related to specific anatomical and functional characteristics. First, hypophysitis can present with headache, and occasionally visual field defects, resulting from pituitary oedema and swelling within the osseous borders of the sela turcica (11, 12, 13, 14, 15). Secondly, acute thyroiditis can result in local thyroid pain, typically alongside symptoms of thyroid hormone excess (16). Whilst CTCAE may accurately reflect these short-term inflammatory symptoms, it less accurately reflects the clinical significance of the hormone deficiencies.

## Hypothalamic–pituitary–adrenal (HPA) axis

### Hypophysitis and hypopituitarism

Pituitary abnormalities are reported in between 1.8 and 18.3% of patients treated with ipilimumab-based regimens (17), usually resulting in panhypopituitarism, associated with inflammatory symptoms such as headache and pituitary enlargement in 50% of cases (13, 14, 15). Hypopituitarism, though not a universal finding (16), usually follows, with ACTH deficiency the most common abnormality, followed by thyroid-stimulating hormone (TSH) and gonadotrophin deficiency (12, 18, 19). We, therefore, propose that hypophysitis should be reserved to describe either the symptomatic phase with headache or MRI findings of enlarged pituitary, whilst ICPI-induced hypopituitarism is used to describe the resultant long-term deficiencies of at least two anterior pituitary hormones (following exclusion of sick euthyroid or sick eugonadal states).

### Isolated ACTH deficiency

Isolated ACTH deficiency (IAD) is induced by PD-1 and PD-L1 inhibition and usually manifests with weakness and loss of appetite without clinical or laboratory or radiological evidence of wider pituitary dysfunction (20, 21). IAD is therefore a clinically distinct pituitary abnormality, caused by specific classes of ICPIs, with different presentations, treatments and prognoses that should be classified separately from hypophysitis and hypopituitarism. IAD presents as hypocortisolism resulting from ACTH deficiency, with intact remaining pituitary axes and preserved aldosterone secretion.

### Adrenalitis

A direct autoimmune damage to the adrenal cortex is relatively rare. This endocrine irAE poses a challenge in terms of evaluating the precise incidence mainly due to the lack of unified criteria for immune-related endocrine adverse effects in clinical trials and also due to incomplete endocrine profiling. Similar to the classic Addison’s disease, depletion of both glucocorticoids and mineralocorticoids yields more pronounced manifestations, including haemodynamic and electrolyte compromise compared to secondary adrenal insufficiency (22, 23, 24). Presentation is with hypocortisolaemia with elevated ACTH and renin levels.

### Posterior pituitary – central diabetes insipidus

Several cases of central diabetes insipidus have been reported so far, presenting with polyuria, polydipsia, hypernatremia, diluted urine with low/absent antidiuretic hormone levels (25, 26).

## Challenges in diagnosing HPA axis endocrinopathies

### Excluding adrenal suppression due to exogenous glucocorticoids treatment

As systemic and topical glucocorticoids are frequently used to treat non-endocrine immune-related toxicity, some patients will develop adrenal suppression. However, due to the risk of multiple toxicities, it is possible that some will also have ACTH deficiency and require long-term glucocorticoid replacement. It is, therefore, important to exclude exogenous glucocorticoid use before making a diagnosis of ACTH deficiency. If exogenous steroids have been used, then standard approaches to weaning should be followed, but if after a prolonged period there has been no recovery of endogenous cortisol production, then the possibility of co-existent ACTH deficiency should be considered. Hence, exclusion on HPA axis suppression by exogenous glucocorticoids should be the first step in the endocrine workup, requiring a careful history to include not just systemic glucocorticoids for the treatment of ICPI-related toxicity, but also topical glucocorticoids such as creams, inhalers or nasal sprays, including those used for non-ICPI-related toxicity (e.g. asthma). Where potentially suppressive doses of corticosteroids have been used (27), a careful weaning protocol should be followed (such as that in Supplementary Appendix 1, see section on supplementary materials given at the end of this article), and a diagnosis of ACTH deficiency should be reconsidered if there is no adrenal axis recovery.

### Replacing single axis dynamic testing with an integrated ‘frozen section’ of the HPA axis

An accurate and rapid diagnosis of an HPA axis autoimmune injury is essential in the context of a cortisol-deficient cancer patient. The distinction between hypophysitis, IAD and adrenalitis has long-term consequences regarding hormone replacement therapy. Whilst hormonal deficiencies require physiological replacement, rare cases of hypophysitis accompanied by severe oedema and pressure on the optic chiasm require treatment with high-dose steroids. A recent study identified an incidence of biochemical hypocortisolaemia in 4.7% of ICPI-treated patients. Using robust endocrine criteria, 14 cases of isolated ACTH deficiency were identified, with 6 of hypophysitis (29), confirming the heterogeneity of presentations, and the need for precise endocrine diagnosis.

Whilst ACTH stimulation tests (Synacthen® test) are used for the assessment of the adrenal glands' stress response, in accordance with the traditional endocrine dynamic diagnostic paradigm, this test is non-discriminative between primary and secondary adrenal insufficiency. A Synacthen® test may demonstrate a rise in cortisol during the first weeks of secondary adrenal insufficiency before adrenal atrophy has occurred. This is of particular concern in ICPI-induced pituitary disorders, given the often rapid onset of cortisol deficiency following checkpoint inhibitors when a Synacthen® test may be falsely reassuring. Therefore, the diagnosis of adrenal insufficiency has to largely rely on the measurement of baseline morning cortisol levels, whilst ACTH and renin levels can help to distinguish the rare cases of primary adrenal insufficiency from those with ACTH deficiency.

Especially where regimens including ipilimumab have been used, a full baseline assessment of other pituitary hormones is required (TSH and free thyroxine (T4), luteinizing hormone, follicle-stimulating hormone, growth hormone, Prolactin, insulin-like growth factor 1, total testosterone for men and oestradiol for pre-menopausal women). Hypocortisolism accompanied by low or inadequately normal ACTH levels with all other anterior pituitary hormones and target glands functioning within the normal range is indicative of IAD. Two or more depleted pituitary axes, reflected by hormone levels below the normal range of target glands (thyroid hormones, etc.) and low or inadequately normal pituitary hormone levels indicate hypopituitarism (with or without hypophysitis), whilst low cortisol and aldosterone levels with compensatory elevation in ACTH levels and in renin levels or renin activity correspond with primary adrenal failure due to adrenalitis.

Notably, this approach may lead to a degree of underdiagnosis in those with partial ACTH deficiency, and in the presence of symptoms compatible with adrenal insufficiency and indeterminate basal cortisol levels, insulin stress testing may be required where there are no contra-indications. Long-term follow-up data will be required to fully elucidate the long-term outcomes in those in this category.

## Management of HPA abnormalities

We propose that assessing patients with HPA axis abnormalities after receipt of ICPI should be focused on two principles:

- Management of any active inflammatory/hypophysitis phase
- Prompt assessment for and replacement of any endocrine deficiencies.

**Figure 1:**
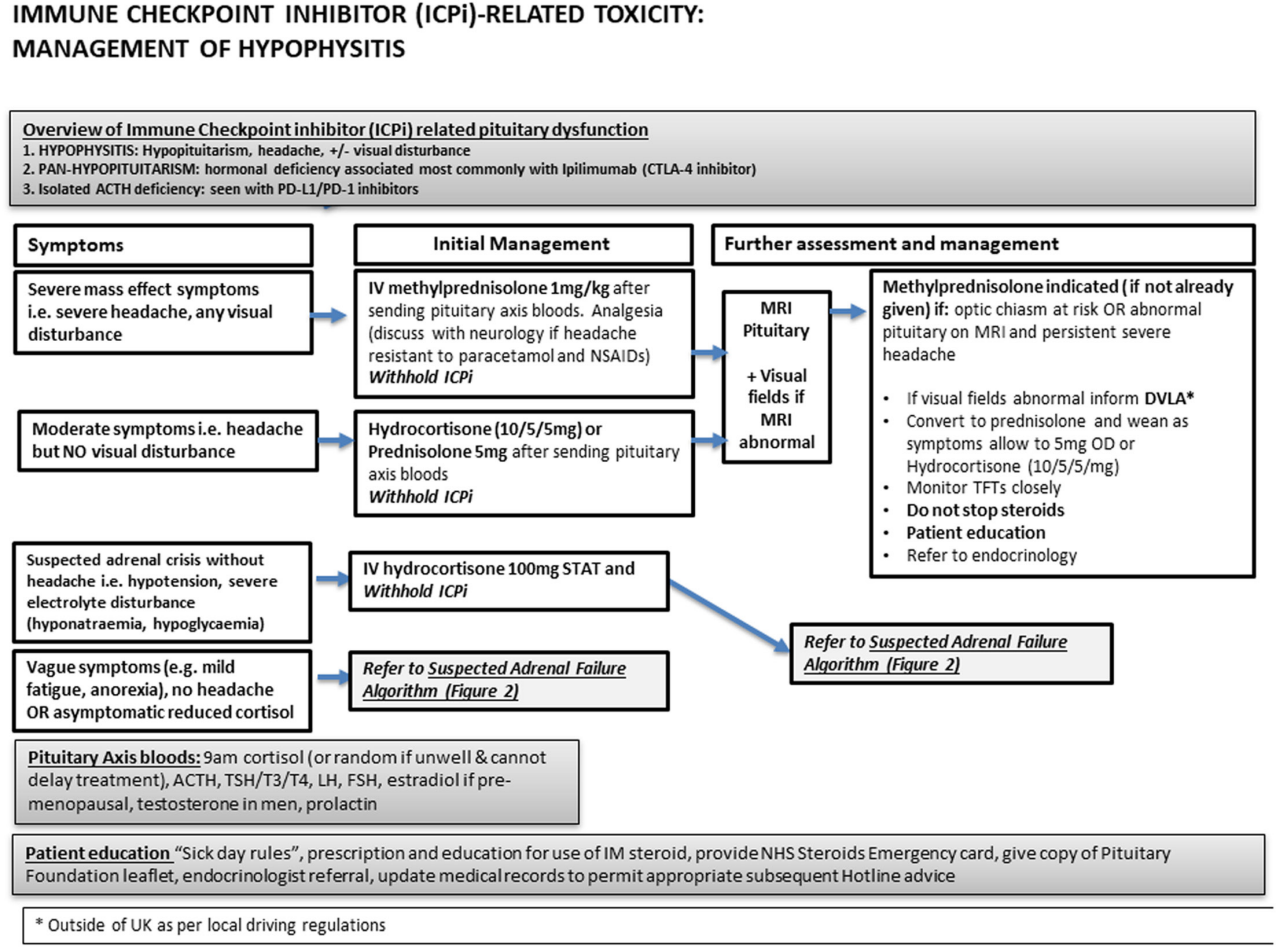
Algorithm for assessment of management of hypophysitis following ICPI.

In those presenting with severe symptoms of hypophysitis, for example, headache, urgent imaging of the pituitary is required, both to confirm pituitary enlargement and exclude other causes such as brain metastases. High-dose corticosteroids in the form of methylprednisolone are only indicated in those with pituitary enlargement that may lead to chiasma compression, as this treatment is associated with poorer oncological outcomes (29), and does not lead to recovery of endocrine function in those with hypopituitarism (30). Oral prednisolone at 30–40 mg daily could be considered if necessary for short-term control of inflammatory symptoms such as headache. An algorithm for assessment of those presenting with symptoms of hypophysitis is shown in Fig. 1.

**Figure 2:**
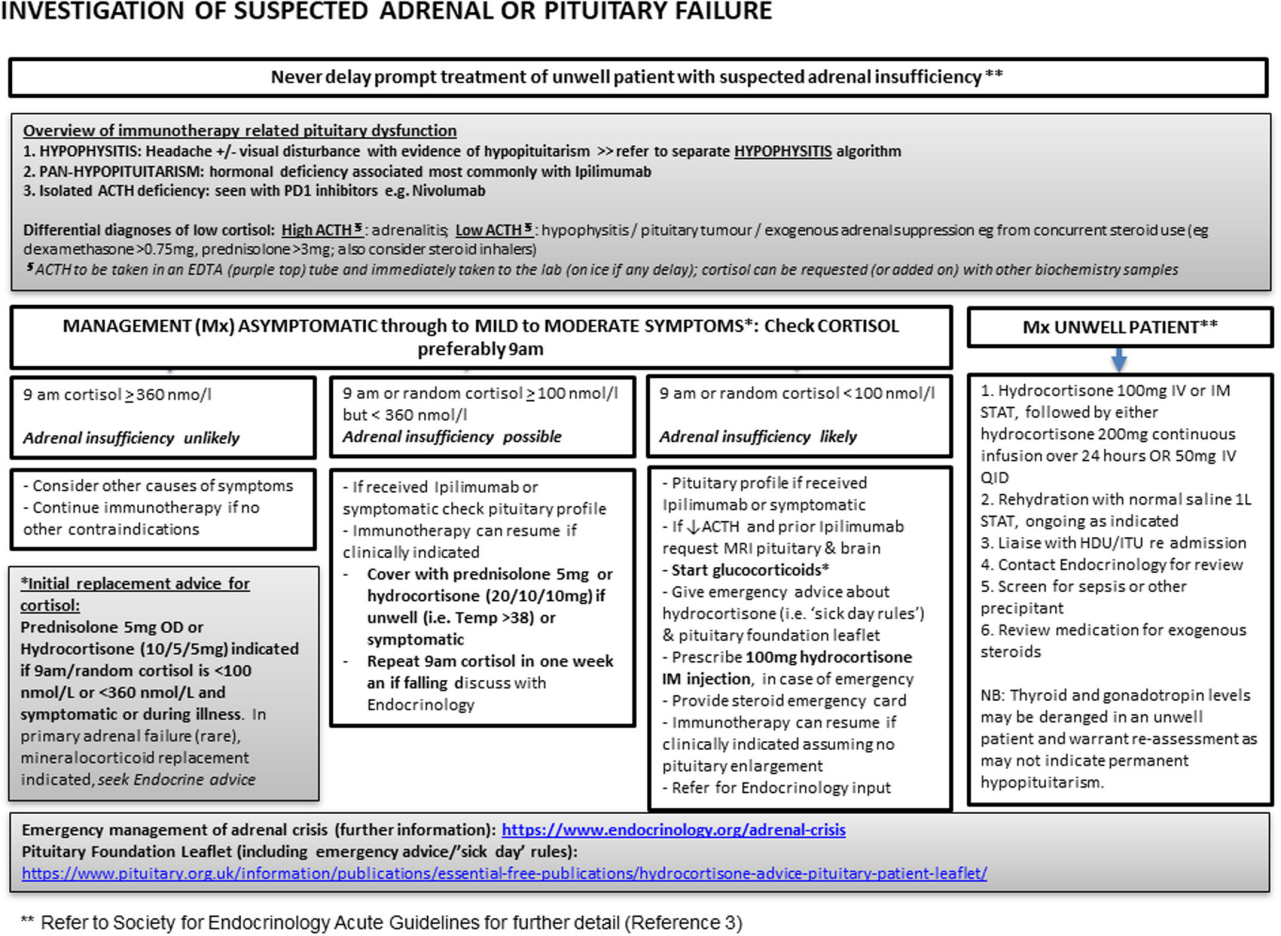
Algorithm for assessment and management of adrenal insufficiency following ICPI.

Those who present acutely unwell or with signs of adrenal crisis should be managed as per standard approaches, as outlined in the Society for Endocrinology guidelines for the acute management of the endocrine complications of checkpoint inhibitor therapy (3). This should not be delayed whilst awaiting imaging even in those with symptoms of hypophysitis. However, there is increasing experience with the use of physiological replacement doses of glucocorticoids (e.g. hydrocortisone 20 mg daily in three divided doses or prednisolone 2–4 mg od) on an out-patient basis in those who are not systemically unwell (31) (Fig. 2). Replacement with levothyroxine and sex hormones may be required as per standard approaches in time, although morning cortisol should always be checked and replacement initiated prior to starting levothyroxine. Whilst there is some evidence of recovery of thyroid and gonadal axis in some patients, necessitating re-testing over time, ACTH deficiency is mostly permanent (19). Although primary adrenal insufficiency is rare, if the ACTH or renin level was elevated then mineralocorticoid replacement with fludrocortisone is required.

## Screening for pituitary abnormalities

Most patients with pituitary abnormalities from ICPI treatment are diagnosed after presenting with symptoms of either hypophysitis hypopituitarism or isolated ACTH deficiency. Although pituitary dysfunction may be detected on routine testing during ICPI therapy, there is little evidence to guide a screening strategy. All available guidelines and summary of product characteristics on ICPI-IE recommend checking thyroid function on each cycle of treatment, and changes in TSH (13) and free T4 (18) have been shown to precede the development of pan-hypopituitarism in those treated with ipilimumab. A fall in early morning cortisol has also been used to detect adrenal insufficiency at an early stage, increasing out-patient treatment (32), but this may be limited by the challenges of early morning testing. Therefore, currently a high index of clinical suspicion for symptoms of either hypophysitis or hypoadrenalism is required.

## Thyroid

Thyroid abnormalities are amongst the most common irAEs described after ICPI therapy (33, 34, 35, 36, 37, 38, 39). Both hyperthyroidism and hypothyroidism occur, sometimes in the same patients. Sub-clinical thyroid abnormalities are even more common, not always requiring therapy (33). Patients with pre-existing thyroid abnormalities (manifested either as the presence of thyroid autoantibodies (40) or higher TSH at baseline (33)) and prior TKI therapy (41) are at increased risk for thyroid dysfunction. Two mechanisms have been proposed – an immune-mediated destructive thyroiditis and Grave’s disease.

### Thyroiditis with transient hyperthyroidism

Thyroiditis is the most common endocrinopathy, affecting 20–30% of ICPI-treated patients. It usually starts a few weeks after the initial introduction to checkpoint inhibitors, with a transient thyrotoxic phase, presumed to reflect spillage of pre-formed hormone, from the inflamed gland. Interestingly, this phase is usually less symptomatic than would be expected in other thyrotoxic states (42). In contrast to Hashimoto's disease, which may evolve at various paces and extents, from a partial, intermittent thyroid insufficiency to complete elimination of thyroid function, the robust cytotoxic activity induced by ICPIs leads to a rapid and complete tissue consumption in the majority of cases. Hence, hypothyroidism usually follows, resulting in a requirement for lifelong thyroid hormone replacement.

Thyroiditis can also present as increased uptake in the thyroid on FDG PET scanning (43).

### Primary hypothyroidism

Of note, hypothyroidism can develop without a preceding thyrotoxic phase. Whilst this may present with symptoms of hypothyroidism, it is more commonly detected on screening, as most protocols recommend testing thyroid function with each cycle of ICPI.

### Graves’ disease

Rarer are reports of Graves’ disease resulting in persistent thyrotoxicosis requiring antithyroid drugs such as carbimazole (44, 45, 46, 47, 48). Graves’ disease should be considered if thyrotoxicosis persists for more than 4 weeks and in those who present with signs of thyroid eye disease. In these cases, we recommend checking TSH receptor antibodies and/or a thyroid uptake scan, and treatment with antithyroid drugs may be required.

The CTCAE criteria for hyperthyroidism and hypothyroidism (Table 1) are based on symptoms, regardless of hormone levels and specific disease or hormonal pattern. Indeed, patients with a typical thyroiditis pattern, with hyper- then hypothyroidism may fall into both criteria over time. Furthermore, sub-clinical changes in thyroid hormone levels are common, may still require treatment, or at least monitoring, and therefore reporting by CTCAE criteria can underestimate the true incidence of thyroid hormone abnormalities, without clearly separating those with Graves’ disease requiring a different treatment paradigm.

## Management of thyroid dysfunction

Hypothyroidism, whether occurring as the first manifestation of ICPI-related thyroid disease or after a period of thyrotoxicosis, is managed as per standard approaches in the management of primary hypothyroidism (49). This is crucial in those with secondary hypothyroidism (see ‘Pituitary’ section). In particular, levothyroxine should be initiated if the free T4 is low or TSH sustained at >10 mIU/L, at an initial dose of 1.6 µg/kg rounded to the nearest 25 µg, unless there are comorbidities such as uncontrolled ischaemic heart disease or atrial fibrillation, or in those over 65 where an initial dose of 25–50 µg daily can be used. If there is any clinical suspicion of adrenal insufficiency, a morning cortisol should be checked prior to initiating levothyroxine, as increased thyroid hormone levels can precipitate an adrenal crisis (50). It may also be appropriate to initiate at a lower dose if a patient presented with both hypothyroidism and signs of ICPI-induced myocarditis. Thyroid hormone replacement is likely to be lifelong, and there is some evidence that ICPI-induced hypothyroidism may require a higher average dose than Hashimoto’s thyroiditis (51).

In contrast, as the hyperthyroid phase of thyroiditis is usually short-lived, treatment is usually supportive. As in other forms of thyroiditis, beta-blockers such as propranolol can be used for symptomatic relief, and occasionally, patients with severe neck pain may require systemic glucocorticoids. There is a limited role for antithyroid drugs. However, in those who present with signs of thyroid eye disease or whose thyrotoxicosis persists for more than 4 weeks, we recommend checking TSH receptor antibodies and/or a thyroid uptake scan, and consider antithyroid drugs such as carbimazole if positive or in persistent thyrotoxicosis, due to the rare occurrence of Graves’ disease. In these rare cases of severe thyrotoxicosis due to immune-related Graves’ disease, it may be necessary to withhold the ICPI, at least until the thyrotoxicosis is controlled, and possibly permanently in those with significant thyroid eye disease.

## Parathyroid

Symptomatic hypocalcaemia accompanied by low levels of parathyroid hormone (PTH) has been described in several case reports (52, 53). ICPI-induced hypoparathyroidism differs mechanistically from the other endocrine irAEs. Activating autoantibodies against the calcium-sensing receptor disrupt the glands’ activity, rather than autoimmune gland destruction (54, 55, 56). This endocrinopathy was shown to be reversible when a patient with concomitant high-grade immune-related colitis received high-dose glucocorticoids (52), assumingly due to improved immune regulation that led to attenuation of activating antibodies to the calcium-sensing receptors. The reversal indicates a controllable disorder in calcium-related communication in the presence of otherwise intact parathyroid glands.

### Management

Initial management is calcium replacement, which may require intravenous calcium infusion, and the use of vitamin D analogues, as per other forms of hypoparathyroidism (57). However, consideration should be given to a trial of glucocorticoids, especially if calcium-sensing receptor antibodies are detected, given the potential reversibility.

## Diabetes and hyperglycaemia

Programmed Death-1 (PD-1)- and Programmed Death-Ligand 1 (PD-L1)-based treatment regimes can result in a rapid onset, insulin-deficient state closely resembling type 1 diabetes, although autoantibodies are detected in less than 50% of cases (6, 9, 58, 59, 60, 61). This results in a need for lifelong insulin treatment, with all the associated complexities. Those with pre-existing type 2 diabetes may also develop this important and potentially life-threatening complication (62, 63). However, a third of patients require high-dose glucocorticoids for non-endocrine reasons and some of these will develop steroid-induced hyperglycaemia (64, 65). Currently, both will be categorised under hyperglycaemia toxicity, with grade determined by the need for intervention or glucose levels that do not match the established levels for diagnosing diabetes, despite the very different mechanisms. Notably, the long-term implications of permanent checkpoint inhibitor-associated insulin-deficient diabetes and other temporary forms of hyperglycaemia, including steroid-induced, are very different, with the former requiring long-term insulin therapy, whilst the latter, representing an indirect side effect, frequently resolves on cessation of the glucocorticoids.

Management is based on a careful assessment of the cause, between steroid-induced hyperglycaemia and immunotherapy induced diabetes, according to standard diabetes approaches to these conditions (66, 67) and a diagnostic approach to safely manage new-onset hyperglycaemia has recently been proposed (65).

## Summary

The current CTCAE classification of endocrine irAEs is based on short-term symptoms rather than accurate diagnostic criteria and the implications of long-term hormone replacement. This makes accurate documentation and reporting of the severity, nomenclature and incidence of endocrinopathies challenging resulting in the under-recognition of largely asymptomatic hormonal abnormalities. This issue, if not addressed in clinical practice, will hamper the development of more evidence-based strategies for investigation and treatment, as well as studies into the longer-term implications of endocrinopathy.

We therefore propose a new classification of endocrine toxicity of ICPI therapy that accurately reflects the pathology, the hormonal disturbance and the treatment (Table 2). We also propose diagnostic criteria, based on an assessment of hormonal function combined with imaging and clinical assessment where appropriate, to enable standardised diagnosis and reporting of endocrine outcomes. This can apply both to endocrine toxicity presenting symptomatically and diagnosed based on laboratory abnormalities detected during screening, as in standard endocrine practice.

**Table 2:** Proposed diagnostic classification and criteria for ICPI-induced endocrinopathy.

Pathology/description	Proposed diagnostic criteria
**Thyroid**	
Thyroiditis – active inflammation, usually clinically silent, rare cases of gland swelling or tenderness. Tissue damage coincides with spillage of preformed thyroid hormones to the bloodstream, manifesting with rapid elevation of free T4 levels and consequent TSH suppression, followed most commonly by subsequent hypothyroidism, or, in rare cases by return to normal thyroid function Post CPIs Hypothyroidism – complete and permanent hypothyroidism following a thyrotoxic phase, in the majority of cases, or as a single-phase thyroid functional decline Graves’ disease – thyrotoxicosis persisting for more than 6 weeks, with elevated free T3, with or without thyroid eye disease or positive thyroid stimulating antibodies	Hyperthyroidism is defined as elevated free T4 or T3 with low or suppressed TSH, in the absence of eye signs of thyroid eye disease, that resolves within 6 weeks, to either euthyroidism or hypothyroidism. Local symptoms of thyroiditis support but are not required for this diagnosis Low free T4 with elevated TSH, with or without a prior thyrotoxic phase Elevated free T4 or T3 with low or suppressed TSH, with either positive TSH receptor antibodies, increased uptake on an isotope scan or co-existent thyroid eye signs, or thyrotoxicosis that persists for more than three months
**Pituitary**	
Hypophysitis – active pituitary inflammation Hypopituitarism – deficiency of two or more pituitary axes, with or without evidence of hypophysitis Isolated ACTH deficiency usually without hypophysitis^a^	Pituitary enlargement on MRI with or without headache, or combination of headache and new onset hypopituitarism (see point 2) Deficiency of two or more pituitary axis: deficiency defined as: 08:00–10:00 h cortisol below assay-specific reference range with non-elevated ACTH, in the absence of exogenous glucocorticoids^b^ Free T4 below reference range with non-elevated TSH Morning testosterone (males) below reference range with non-elevated gonadotrophins on more than one occasion Secondary amenorrhea with oestradiol < 100 pmol/L and non-elevated gonadotrophins (females pre-menopause) Prolactin above or below reference range can support a diagnosis 09:00 h cortisol below assay specific reference range with non-elevated ACTH, in the absence of exogenous glucocorticoids^b^ Other pituitary axis intact
**Adrenal**	
Primary adrenal insufficiency – cortisol deficiency with either elevated ACTH or renin > 2 × ULN	08:00–10:00 h cortisol below assay-specific reference range with elevated ACTH or renin > 2 ULN
**Posterior pituitary**	
Central diabetes insipidus – new onset polyuria and polydipsia	24-h urine volume greater than 50 mg/kg body weight; water deprivation test revealing diluted urine (osmolality below 100) when serum osmolality exceeds 295 mOsm/kg, followed by a desmopressin challenge leading to urine concentration above 300 mOsm/kg
**Endocrine pancreas**	
New-onset hyperglycemia Diabetic ketoacidosis	Blood glucose measurements > 11.1 mmol/L; 200 mg/dL in the absence of steroids treatment in two or more occasions after immunotherapy with C-peptide <100 pmol/L when available^c^ positive islet cell/IA2/GAD antibodies’ titre are supportive but not mandatory New onset hyperglycaemia as above with pH < 7.30 and/or bicarbonate < 15.0 mmol/L and capillary or blood ketones > 3.0 mmol/L
**Parathyroid**	
New onset hypocalcemia Activating antibodies to the calcium-sensing receptor (CaSR) , of IgG1 and IgG3 subclasses with affinity to functional epitopes on the receptor, thus causing hypocalcaemia	Albumin corrected calcium below 2.1 mmol/L with low PTH levels and normal magnesium levels

^a^Exclude use of exogenous glucocorticoids prior to diagnosing Isolated ACTH deficiency, reconsider if failure of adrenal recovery after the standard withdrawal approach; ^b^In those with ongoing symptoms of adrenal insufficiency but 09:00 h cortisol within the reference range, consider an insulin tolerance test to confirm or rule out HPA axis dysfunction if no contra-indications; ^c^In those treated with high-dose glucocorticoids, without ketosis, steroid-induced hyperglycemia should be considered.

Expected benefits from the new diagnostic system:

1. More accurate documentation and reporting of endocrinopathies.
2. Appropriate clinical management based on long-term perspective regarding hormone replacement therapy rather than short-term symptoms.
3. Prevention of unnecessary cessation or interruption of ICPI therapy and unnecessary corticosteroid therapy.
4. Improved reporting and future development of trial protocols, as endocrinopathies already present an exception, where current guidelines support the continuation of ICPI, having initiated hormone replacement therapy, regardless of the grade. The proposed classification would make the development of guidelines easier as the treatment would more clearly reflect the underlying diagnosis.
5. Enhanced confidence in clinical diagnostic coding (by applying these criteria when formalising diagnosis) to facilitate improved accuracy in determining incidence, prevalence, and outcomes in prospective and retrospective real-world data analysis. Future audits of clinical practice will also be refined by clearer diagnostic criteria and development of specific diagnostic codes for ICPI-IEs. To support this, and to facilitate future audits, Table 3 contains suggested use of SNOMED codes to document the different endocrine IRAEs in electronic health records in a standardised manner. This classification will allow the identification of those with IRAEs whilst also allowing linkage to standard endocrine diagnostic terms.

**Table 3:** Recommended SNOMED codes for recording Endocrine IRAEs (based on United Kingdom Edition v20220803 (SNOMED CT - Home; https://termbrowser.nhs.uk/?)

Term	Suggested use
109954009 |Effects of immunotherapy (finding)|	This term should be used in all individuals with an endocrine adverse event from cancer immunotherapy. This will enable case finding of endocrine IRAEs whilst allowing the use of standardised endocrine terminology.
**Pituitary–adrenal axis disorders**	
237705001 |Hypophysitis (disorder)|	Used in those with clinical or radiological evidence of pituitary inflammation
237698002 |Iatrogenic hypopituitarism (disorder)|	Used in those with two or more anterior pituitary hormone deficiencies
80599001 |Isolated corticotropin deficiency (disorder)|	Where ACTH deficiency is only hormonal abnormality
237763005 |Iatrogenic adrenal insufficiency (disorder)|	Primary adrenal failure
45369008 |Neurohypophyseal diabetes insipidus (disorder)|	Central diabetes insipidus
**Thyroid disorders**	
61556008 |Iatrogenic thyroiditis (disorder)|	Transient hyperthyroidism ± subsequent hypothyroidism
88273006 |Iatrogenic hypothyroidism (disorder)|	Primary hypothyroidism with or without prior thyroiditis
353295004 |Graves' disease (disorder)|	Thyrotoxicosis meeting criteria for Graves’ diagnosis as per Table 2
**Endocrine pancreas**	
408540003 |Diabetes mellitus caused by non-steroid drugs (disorder)|	New onset hyperglycaemia (see Table 2)
420422005 |Ketoacidosis due to diabetes mellitus (disorder)| in addition	DKA (see Table 2)
**Parathyroid**	
36976004 |Hypoparathyroidism (disorder)|	Hypocalcaemia/hypoparathyroidism

We propose the adoption of these new diagnostic criteria for ICPI-induced endocrine dysfunction that recognise their unique characteristics compared to other forms of irAEs and to classic endocrine diseases. The revised criteria would enable accurate assessment of different endocrinopathies, matching the required treatment, and allow care planning to occur, alongside existing management guidelines (4, 68).

## Supplementary Material

supplementary Material

## Declaration of interest

DM reports personal fees from Bristol Meyer Squibb, personal fees from MSD, personal fees from Roche. SC reports personal fees from Bristol Myer Squibb. RP, SA and KY report no declarations.

## Funding

This study did not receive any specific grant from any funding agency in the public, commercial or not-for-profit sector.
