# Supplementary material for: Diagnostic criteria and proposed management of immune-related endocrinopathies following immune checkpoint inhibitor therapy for cancer
Source: Endocr Connect. 2023 Apr 17;12(5):e220513. doi: 10.1530/EC-22-0513 (PMC10160541; doi:10.1530/EC-22-0513)
Supplement: supplementary Material [file supplementary_material.pdf]

DRAFT STEROID WITHDWAL PROTOCOL

1. Has patient received glucocorticoids at a dose equivalent to >5mg prednisolone for more than 4 weeks?

Have they been clinically stable on prednisolone 5mg or (or equivalent) less and has medical team *confirmed that steroids are no longer required for disease modification of the primary medical condition'?*

Was there a prior diagnosis of hypophysitis, ACTH deficiency or adrenal failure? If YES REDUCE PREDNISOLONE TO 4mg od and refer back to treating endocrinologist. For patients with no prior history of adrenal insufficiency, follow steps 2 to 5 below.

2. Once taking prednisolone 5mg od or less

ENSURE STILL HAS STEROID CARD

SICK DAY RULES (SEE LEAFLET)

Consider EMERGENCY HYDROCRTISONE INJECTION

Check 9am cortisol BEFORE TAKING MORNING DOSE OF STEROIDS

<200 nmol/L reduce prednisolone dose by 0.5-1mg / month retesting every 2 months

>200 but less than 360 – arrange short synacthen test

Pass (Peak > 400 nmol/L and increment > 170 nmol/L) – STOP STEROIDS

Fail – reduce prednisolone as above

>360 – STOP STEROIDS

3. Once been on 3mg od for 1 month repeat 9am pre dose cortisol

If patient symptomatic (eg fatigue, dizziness consider slower withdrawal eg reduce by 1mg every 6-8 weeks).

<100 – continue prednisolone 3mg od for further 2 months then re-test.

>100 but <200 - reduce prednisolone to 2mg of for 1 month then 1mg od for 1 month and repeat step 4

>200 but less than 360 – arrange short synacthen test

Pass (Peak > 400 nmol/L and increment > 170 nmolL) – STOP STEROIDS

Fail – reduce prednisolone to 2mg of for 1 month then 1mg od for 1 month and repeat step 4

>360 – STOP STEROIDS

5. If cortisol still low or fails SST after prednisolone 1mg for 1 month refer to endocrinology - ? silent ACTH deficiency
